# DeepGlioSeg: advanced glioma MRI data segmentation with integrated local-global representation architecture

**DOI:** 10.3389/fonc.2025.1449911

**Published:** 2025-02-04

**Authors:** Ruipeng Li, Yuehui Liao, Yueqi Huang, Xiaofei Ma, Guohua Zhao, Yanbin Wang, Chen Song

**Affiliations:** ^1^ Department of Urology, Hangzhou Third People’s Hospital, Hangzhou, China; ^2^ College of Medical Technology, Zhejiang Chinese Medical University, Hangzhou, China; ^3^ Department of Psychiatry, Hangzhou Seventh People’s Hospital, Hangzhou, China; ^4^ Department of Magnetic Resonance Imaging, First Affiliated Hospital of Zhengzhou University, Zhengzhou, China

**Keywords:** automated segmentation, glioma, CTPC, convolutional neural network, magnetic resonance imaging

## Abstract

**Introduction:**

Glioma segmentation is vital for diagnostic decision-making, monitoring disease progression, and surgical planning. However, this task is hindered by substantial heterogeneity within gliomas and imbalanced region distributions, posing challenges to existing segmentation methods.

**Methods:**

To address these challenges, we propose the DeepGlioSeg network, a U-shaped architecture with skip connections for continuous contextual feature integration. The model includes two primary components. First, a CTPC (CNN-Transformer Parallel Combination) module leverages parallel branches of CNN and Transformer networks to fuse local and global features of glioma images, enhancing feature representation. Second, the model computes a region-based probability by comparing the number of pixels in tumor and background regions and assigns greater weight to regions with lower probabilities, thereby focusing on the tumor segment. Test-time augmentation (TTA) and volume-constrained (VC) post-processing are subsequently applied to refine the final segmentation outputs.

**Results:**

Extensive experiments were conducted on three publicly available glioma MRI datasets and one privately owned clinical dataset. The quantitative and qualitative findings consistently show that DeepGlioSeg achieves superior segmentation performance over other state-of-the-art methods.

**Discussion:**

By integrating CNN- and Transformer-based features in parallel and adaptively emphasizing underrepresented tumor regions, DeepGlioSeg effectively addresses the challenges associated with glioma heterogeneity and imbalanced region distributions. The final pipeline, augmented with TTA and VC post-processing, demonstrates robust segmentation capabilities. The source code for this work is publicly available at https://github.com/smallboy-code/Brain-tumor-segmentation.

## 1 Introduction

Brain tumors, also known as intracranial tumors in medical terminology, are abnormal masses of tissue characterized by uncontrolled cell growth and proliferation. According to the National Brain Tumor Society (1), gliomas account for approximately one-third of all brain tumors. Gliomas predominantly originate from glial cells, which surround and support the neurons in the cerebral cortex. These glial cells include ependymal cells, oligodendrocytes, and astrocytes. Gliomas put pressure on the brain or spinal cord, causing symptoms such as headaches, changes in personality, and weakness in the arms, etc. (2). They can disrupt brain function and pose a significant threat to an individual’s life. The exact cause of gliomas remains unclear, and they can develop in all age groups, with a higher incidence observed in adults. Early detection and diagnosis of gliomas are critical to the effectiveness of treatment. Therefore, it is important to identify and diagnose gliomas in a timely manner to improve therapeutic outcomes.

**Figure 1:**
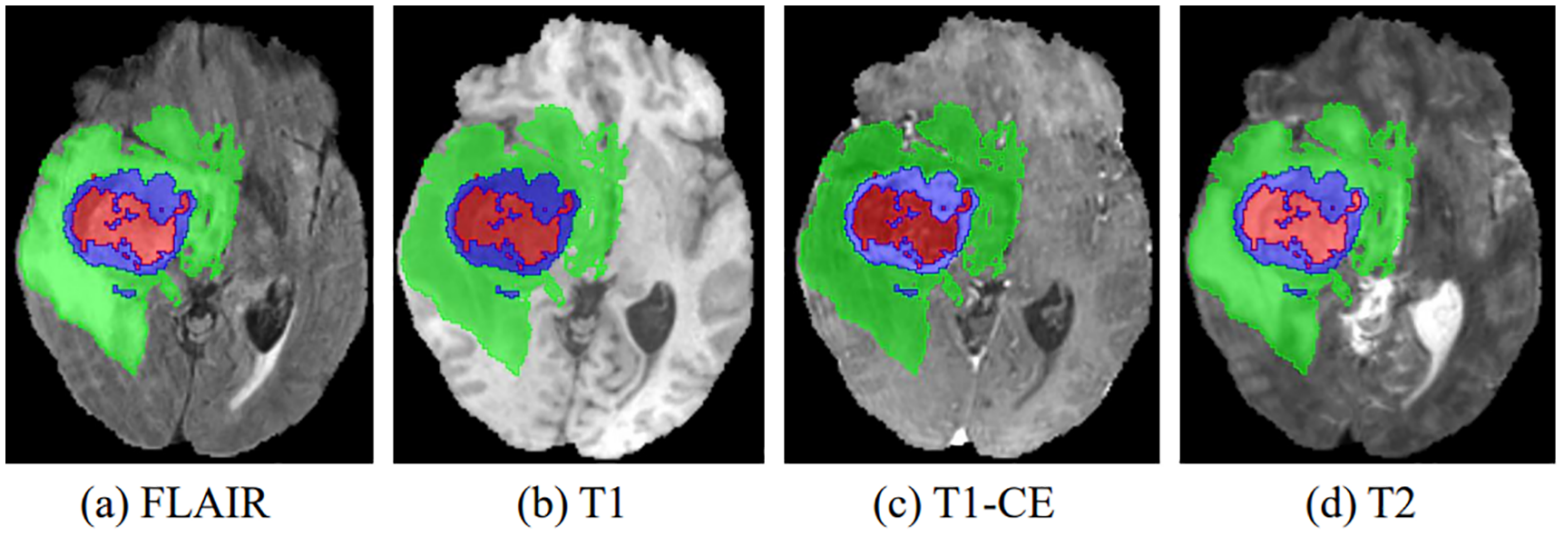
The four multimodal MR images with glioma tumors are: **(A)** FLAIR, **(B)** T1, **(C)** T1-CE, and **(D)** T2. Different colors are used to differentiate tumor subregions: red for necrotic and non-enhanced tumor (NCR/NET), green for peritumoral edema (ED), and blue for enhanced tumor (ET).

In recent years, advances in medical imaging techniques such as positron emission tomography (PET), computed tomography (CT), and magnetic resonance imaging (MRI) have become increasingly important in the detection and diagnosis of disease. These different imaging modalities have the ability to identify distinct tumor regions within soft tissue (3). Typically, gliomas can be identified using a variety of MRI modalities, including T1-weighted (T1), T1-weighted with contrast enhancement (T1-CE), T2-weighted (T2), and T2-weighted fluid-attenuated inversion recovery (FLAIR). Each of these imaging modalities offers unique perspectives and insights into the properties of the tumor, resulting in different representations of the tumor on the images, as shown in **Figure 1**. After acquiring multimodal volumetric data of gliomas, a meticulous pixel-by-pixel segmentation process is applied to each individual slice until the entire 3D brain volume is accurately delineated into informative areas, establishing the ground truth (GT). The resulting segmentation output is then central to subsequent stages, including diagnosis, treatment planning, surgical strategies, and ongoing monitoring of tumor dynamics and changes.

In the search for valuable insights into brain tumors, radiologists have traditionally relied on manual segmentation of MRI images, using their expertise in anatomy and physiology (4). However, manual pixel-level segmentation of brain tumors by radiologists is a labor-intensive process (5). Radiologists face significant challenges during manual segmentation due to factors such as indistinct boundaries of gliomas, which includes peritumoral edema, necrotic cores, and tumor core enhancement. As a result, manual segmentation efforts by radiologists typically yield Dice scores in the range of 74% to 85% (6). Furthermore, manual segmentation is time-consuming, with radiologists spending 3–5 hours annotating an MRI scan for a single patient (7). Therefore, fully automated glioma segmentation methods are of paramount clinical importance and practical value (8).

Glioma segmentation faces significant challenges characterized by high heterogeneity and regional imbalances. First, high heterogeneity is evident in the wide variety of tumor shapes, structures, and locations. As shown in **Figure 1**, gliomas exhibit considerable inter-patient variability in structural characteristics, geometric configurations, and spatial distributions. This inherent variability poses a significant impediment to the accuracy of glioma segmentation. Consequently, an optimal model must effectively capture both local features (such as texture and edges) and global features (including shape, location, and structure) of gliomas. However, most existing convolutional neural networks (CNNs) focus primarily on extracting features at the local level, falling short of achieving a comprehensive representation.

Second, regional imbalance arises from the large size differences between the brain tumor, the background, and various tumor subregions. In the case of the BraTS2020 dataset, pixels within the tumor region represent only 1.1% of the total pixels. This tiny fraction of the tumor region may inadvertently cause the model to prioritize the background region, hindering accurate characterization of tumor features. Moreover, the proportions of each tumor subregion within the total tumor are significantly different (58%, 19.8%, and 22.2% for the whole tumor (WT), enhanced tumor (ET), and tumor core (TC), respectively). This unbalanced distribution among subregions presents a substantial challenge for the model in classifying categories within these smaller proportions.

Many existing methods incorporate global information to address the challenges mentioned above. Typically, these methods use atrous convolution to expand the receptive field. However, in scenarios involving data types with smaller regions, such as brain tumors, atrous convolution may miss pixels, making it less suitable. A limited number of methods have used self-attention mechanisms to establish long-range dependencies. For example, Chen et al. (9) introduced a Parallel Self-Attention (PSSA) mechanism that transforms self-attention into a standard convolution operation on an appropriately transformed feature. This innovation effectively unifies self-attention and convolution. However, this approach diffuses local features into global features through layer stacking, which may dampen the performance of the method. Notably, the Transformer architecture excels at capturing global representations and requires fewer computational resources compared to traditional self-attention mechanisms (10). For example, Zhang et al. (11) proposed the parallel branched TransFuse network, which combines both Transformer and CNN architectures. This network includes a BiFusion module, consisting of spatial attention and channel attention, to facilitate feature fusion between the two branches. However, a limitation of this approach is the lack of fusion between the Transformer and CNN branches during the down sampling process, as these branches remain independent.

**Figure 2:**
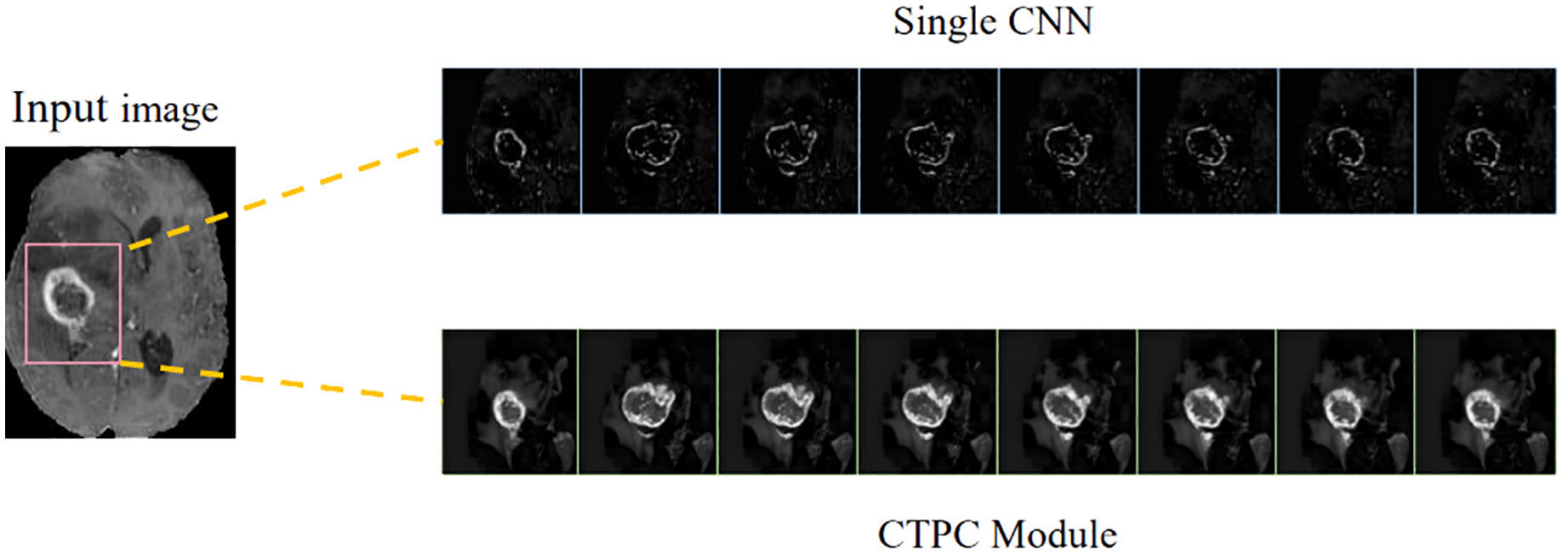
Comparison of feature maps between the CTPC module and CNN.

In the present study, we develop a unique DeepGlioSeg framework that enables glioma segmentation in multimodal MRI data. This network adopts a U-shaped architecture with skip connections, strategically used to support the continuous exploitation of contextual information. The DeepGlioSeg network introduces a central CTPC (CNN-Transformer Parallel Combination) module as its core component, comprising parallel branches for both CNN and Transformer networks. This innovative module facilitates the fusion of local and global features within glioma images through the collaborative interaction of these two branches. As a result, it effectively captures both the global and local features of gliomas, mitigating the challenges posed by the high heterogeneity in tumor shape, structure, and location. As shown in **Figure 2**, this module consistently outperforms convolution-based feature maps in accurately capturing the intricate shape and structure of gliomas. In addition, the DeepGlioSeg network employs a weighted loss approach to address the issue of region imbalance. It extends the generalized Dice loss to account for multiple regions and adjusts the contribution of each region with weighted values. Specifically, larger loss weights are assigned to categories associated with smaller regions, thereby increasing the focus on the tumor region.

We summarize our contributions as follows:

1. Our method introduces a CTPC module, which includes both a Transformer branch and a parallel CNN branch. This module facilitates the fusion of local and global features within glioma images through the interactive cooperation of these two branches, enhancing contextual relationships.
2. Our method assigns specific weights to each region based on the volume ratio of the region relative to the background. This weighting mechanism increases attention to the tumor region.
3. To evaluate the robustness of our algorithm, we curated a private brain tumor dataset consisting of data from 232 patients. Extensive experiments were performed on this private dataset, as well as on three publicly available datasets. The results consistently demonstrate the effectiveness of our proposed approach.

## 2 Related works

### 2.1 Brain tumor segmentation methods

Previous research on brain tumor segmentation in MR images can be categorized into (1) machine learning-based segmentation methods and (2) CNN-based segmentation methods. Machine learning-based methods have been adapted for brain tumor segmentation tasks, such as support vector machines (SVM) (12) and random forests (13). For example, Bauer and colleagues (14) employed SVM classification methods in conjunction with hierarchical conditional random field regularization to improve the segmentation of brain tumor images.

CNNs have been extensively applied to brain tumor segmentation tasks, yielding remarkable results. For instance, spatial attention gates and channel attention gates were introduced into the U-Net network architecture by Xu et al. (15). Additionally, Xu et al. (15) developed a new FCN with a feature reuse module and a feature integration module (F2-FCN), enabling the extraction of more valuable features by reusing features from different layers. Shen and Gao (16) introduced a network with an encoding path that operates independently across channels and a decoding path focusing on feature fusion. This network utilizes self-supervised training and presents a novel approach to domain adaptation on the feature map, mitigating the risk of losing important restoration information within channels.

Cascaded methods have also emerged as a key research focus in brain tumor segmentation, achieving notable advancements through various strategies. For instance, Le Folgoc et al. (17) introduced lifted auto-context forests, a multi-level decision tree structure that optimizes segmentation via auto-context mechanisms. Wang et al. (18) proposed a cascaded anisotropic convolutional neural network, enhancing tumor edge and structure segmentation with anisotropic convolutional kernels. Lachinov et al. (19) iteratively refined segmentation results using a cascaded 3D U-Net variant, demonstrating its efficacy on the BraTS2018 dataset. Weninger et al. (20) designed a two-step approach with a 3D U-Net for tumor localization, followed by another for detailed segmentation into core, enhanced, and peritumoral edema regions. Finally, Ghosal et al. (21) developed a deep adaptive convolutional network with an adaptive learning mechanism that dynamically adjusts parameters, addressing the complexity of multimodal MRI.

### 2.2 Segmentation combined with CNN and transformer

The application of Transformer architecture to image segmentation has recently gained prominence, particularly in its integration with CNNs, a fusion that has yielded remarkable results in the field of medical image segmentation (9, 22–24). For example, Cao et al. (22) constructed a Transformer-based U-type skip connection encoder-decoder architecture called Swin-Unet. It is the first pure Transformer segmentation network and successfully demonstrates the applicability of transformers in the visual data domain. Building on Swin-Unet, more and more methods have begun to explore the fusion of Transformer and CNN. For instance, Hatamizadeh et al. (23) presented the architecture of UNet Transformer (UNETR), which uses a pure Transformer as the backbone for learning features in the encoding part, while only CNN is used in the decoding part.

Furthermore, not limited to Transformer, there has been increasing exploration of the application of self-attention mechanisms. For example, Chen et al. (9) theoretically derived a global self-attention approximation scheme that approximates self-attention by performing convolution operations on transformed features. Building on this, some approaches have developed multi-module structures that combine convolution and self-attention to integrate both local and non-local interactions. For instance, Petit et al. (24) presented the U-transformer model, which combines the U-type image segmentation structure with the self-attention and cross-attention mechanisms of the Transformer.

Recent advancements in hybrid CNN-Transformer architectures have significantly improved glioma segmentation by enhancing boundary precision and integrating local and global features. Gai et al. (25) proposed RMTF-Net, which combines ResBlock and mixed transformer features with overlapping patch embedding and a Global Feature Integration (GFI) module to improve decoding quality. Zhu et al. (26) developed a multi-branch hybrid Transformer that combines the Swin Transformer for semantic extraction and a CNN for boundary detection, incorporating a Sobel-based edge attention block to enhance tumor boundary preservation. Hu et al. (27) introduced ERTN, a dual-encoder model with a rank-attention mechanism to prioritize key queries, balancing performance and efficiency. These studies showcase diverse strategies for leveraging CNN-Transformer hybrids to address segmentation challenges, particularly in cases with complex tumor boundaries.

### 2.3 Category imbalance

A common problem in pixel-level semantic segmentation is class imbalance. This issue tends to reduce accuracy in regions belonging to the minority class (28, 29). For example, Hossain et al. (30) suggested that an effective way to address class imbalance is to adjust the loss function. They propose the bifocal loss function (DFL) to correct the problem of vanishing gradients in focal loss (FL). They introduce a regularization term to impose constraints on the negative class labels, which increases the loss for classes that are difficult to classify. Bressan et al. (31) used pixel-level weights in the training phase to dynamically adjust the importance of individual pixels, either increasing or decreasing their weight as needed. In other words, the contribution of each pixel in the loss function is weighted, which increases the importance of minority class pixels. Pan et al. (32) also faced the challenge of unbalanced foreground and background voxels when performing coronary segmentation. They use the concept of focal loss to optimize the network and achieve good results. To address the significant class imbalance problem observed in brain tumors, we follow the approach of the GDL loss function and assign more weight to small class regions, minimizing the model’s focus on background regions.

## 3 Methodology

**Figure 3:**
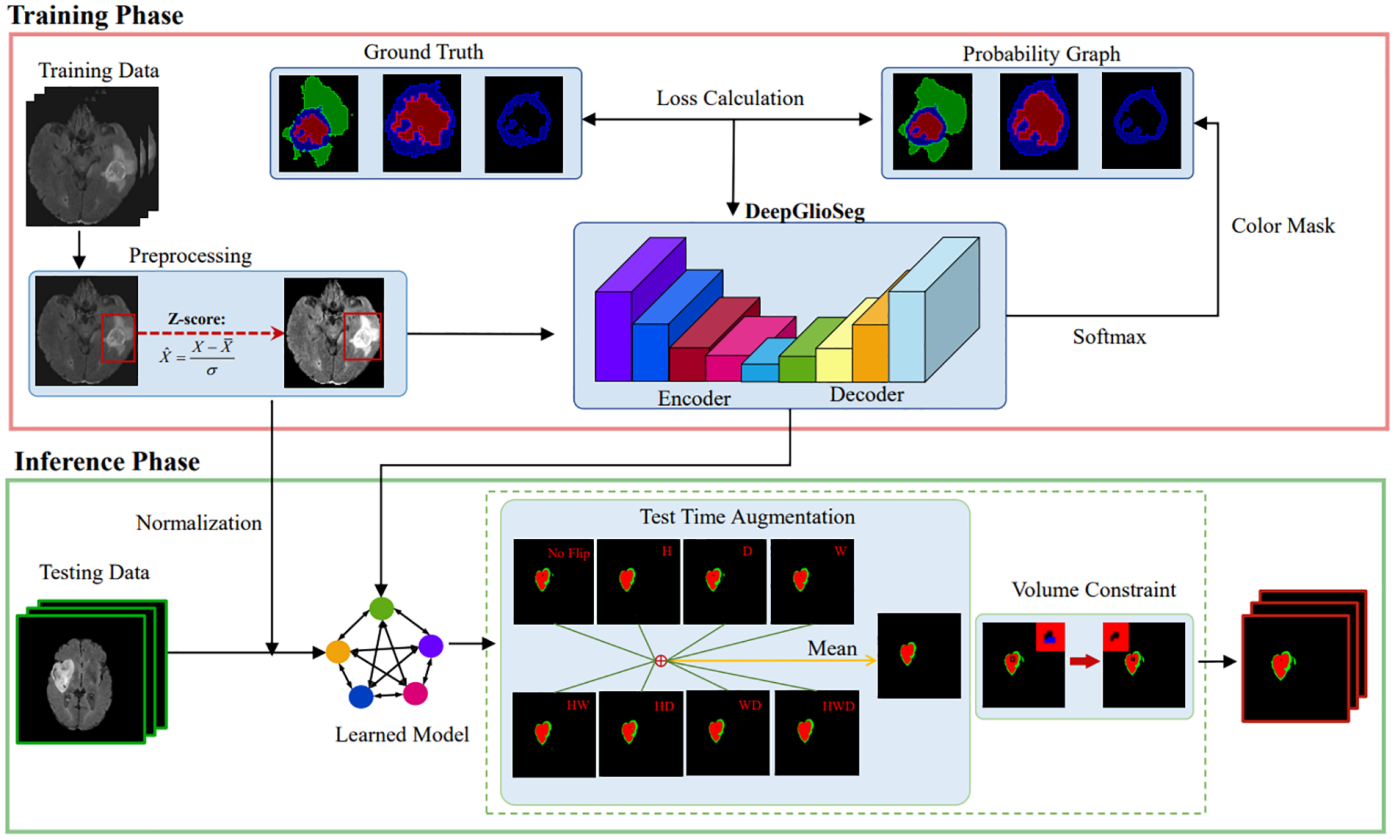
The diagram of the proposed DeepGlioSeg for automated glioma segmentation in multimodal MRI images.

The proposed DeepGlioSeg framework consists of two phases: (1) the training phase, which includes data preprocessing, loss calculation, and parameter updating, and (2) the inference phase, which includes data preprocessing, learned model import, and postprocessing. A diagram summarizing this framework is shown in **Figure 3**.

### 3.1 Preprocessing

We used the BraTS and ZZH datasets, where each brain MRI scan includes FLAIR, T1, T1-CE, and T2 modalities, each with distinct eigenvalue distributions due to contrast differences. These variations pose challenges such as slower convergence and overfitting due to inconsistent intensity scales across modalities. To address these issues, we normalized voxel values within brain regions by subtracting the mean and dividing by the standard deviation. This standardization facilitated effective learning and mitigated convergence issues. Voxel values in non-brain regions were set to zero to eliminate interference from irrelevant background data. The normalization formula is as follows:


(1) $$X^{\prime }=\frac{X-\mu_{B}}{\sigma_{B}}$$

where $X^{\prime }$ represents the processed image, X symbolizes the original voxel value in the brain region, $\mu_{B}$ signifies the average intensity value of the brain region, and $\sigma_{B}$ indicates the standard deviation of the brain region. This approach ensures that the model can focus on meaningful information while reducing variability caused by background noise. To further enhance robustness and generalization, we applied data augmentation techniques such as random rotations, flips, and elastic deformations. These augmentations prevent overfitting by exposing the model to diverse variations, improving its performance on unseen data in real-world clinical settings.

Four sets of modal sequences, each with a size of 240×240×155, were merged to obtain 4-channel 3D image data with a size of 240×240×155×4. Each training example has a corresponding label with a size of 240×240×155. The labels consist of four categories: background (label: 0), necrotic and non-enhanced tumor (label: 1), peritumoral edema (label: 2), and GD-enhanced tumors (label: 4). Finally, based on hardware and computational considerations, a training patch with a size of 128×128×128 was extracted from the training case.

### 3.2 DeepGlioSeg network architecture

**Figure 4:**
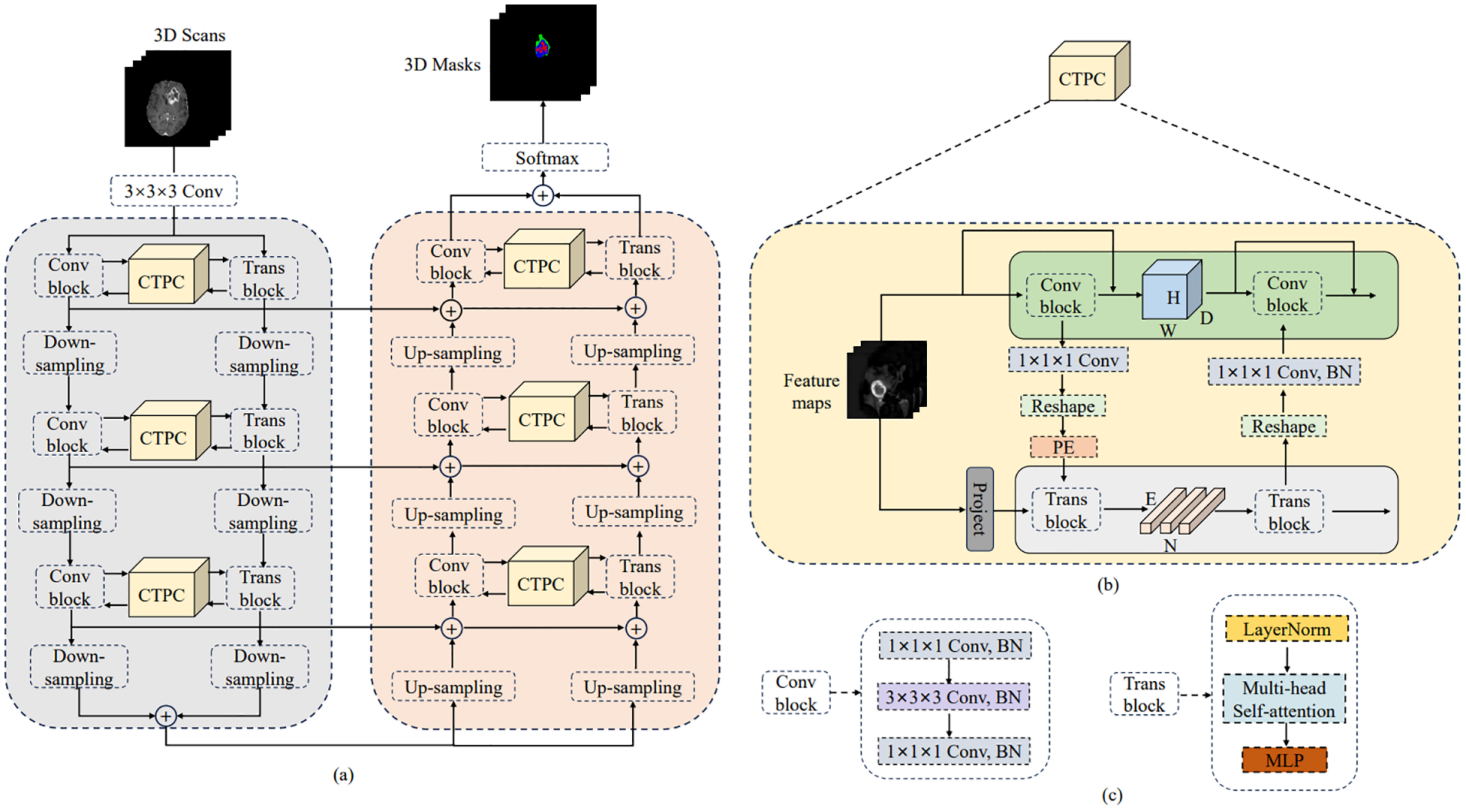
Overview of the proposed DeepGlioSeg network architecture: **(A)** The architecture of DeepGlioSeg. **(B)** The specific implementation steps of the CTPC module. **(C)** The detailed composition of the Conv block and Trans block.

The general design of DeepGlioSeg is shown in **Figure 4A**, which features a symmetric encoder-decoder architecture with skip connections. The basic concept revolves around the alternating stacking of CTPC modules and down sampling layers, combining local features with global representations at different resolution levels. Importantly, the CTPC module maintains consistent feature map sizes, while deconvolution gradually restores resolution. Throughout the network, all convolutional layers are complemented by batch normalization layers and ReLU activation functions. To mitigate overfitting, an initial convolutional layer with dropout functionality is included at the beginning of the model. Additionally, eight successive convolutional layers are implemented at the base of DeepGlioSeg to enhance feature extraction.

To manage the computational demands within the Transformer branch, the Feature Fusion Pathway (FFP) employs different down sampling steps corresponding to various resolution levels while maintaining a patch embedding size of 4096. It is important to note that the feature map input to the Transformer remains constant at 16×16×16.

### 3.3 CTPC module structure

In deep learning, CNNs collect local features at different resolutions by applying convolutional operations, effectively preserving local details as feature maps. Vision Transformers, on the other hand, are specifically designed to aggregate global representations by iteratively processing compressed patch embeddings through a series of self-attention modules. The CTPC module, as shown in **Figure 4B**, consists of three essential elements: the CNN module, the Transformer branch, and the FFP. These components are integrated to facilitate feature fusion between the two branches, effectively enhancing the feature extraction capabilities of the network.

#### 3.3.1 CNN branch

As shown in **Figure 4C**, the CNN branch consists of two iterative convolution modules. Each module contains a sequence of a 1×1×1 downward convolution layer, a 3×3×3 spatial convolution layer, a 1×1×1 upward convolution layer, and a residual link connecting the module’s input and output. While the Vision Transformer encodes image patches into word vectors, potentially leading to a loss of local detail, the CNN branch operates differently. In a CNN, the convolutional kernel glides over the neighborhood map, enabling it to extract continuous local features. This feature allows for the preservation of intricate and detailed local features to a significant extent. As a result, the CNN branch serves as a continuous supplier of local detail to the Transformer branch.

#### 3.3.2 Transformer branch

The Transformer branch includes a multi-head self-attention module and a multi-layer perceptron (MLP) block, as shown in **Figure 4C**. Layer normalization is applied before both the multi-head self-attention module and the MLP block. Additionally, two residual connections are incorporated at corresponding positions. To balance computational efficiency and feature map resolution, the CNN branch output is down sampled to a 16×16×16 patch embedding.

#### 3.3.3 Feature fusion path

The FFP functions to connect and align the shape disparity between the feature map in the CNN pathway and the patch embedding in the Transformer pathway. It actively promotes the continuous integration of local features with global representations through interactive mechanisms. Notably, the shape of the feature stream differs between the CNN and Transformer pathways. Specifically, the CNN feature map has a shape of *C*×*H*×*W*×*D*, where *C*, *H*, *W*, and *D* denote the channel, height, width, and depth, respectively. In contrast, the patch embedding takes the form *E*×*C*, where *E* is the embedding size and *C* is the number of image patches. Prior to inputting the feature map into the Transformer branch, channel alignment of the feature map and patch embedding is achieved by a 1×1×1 convolution. The volume dimensions are then compressed to 16×16×16 using the down sampling module, with different steps chosen for different resolution levels. Finally, the patch embedding is obtained via a reshape operation.

#### 3.3.4 Position embedding

To capture essential positional information crucial for the segmentation task, we introduced learnable positional embeddings that are merged with the patch embedding by direct addition. When transitioning from the Transformer branch back to the CNN branch, it is necessary to upsample the patch embedding to restore it to the original shape of the CNN feature map. A 1×1×1 convolutional layer is then applied to harmonize the channel dimensions. Finally, the resulting output is combined with the feature map. Throughout this process, batch normalization is used to regulate the features.

### 3.4 *Loss f*unction

There is a significant data imbalance between tumor and non-tumor tissue for the purpose of identifying and delineating brain tumors and their subregions. Sudre et al. (33) noted that as the degree of data imbalance increases, the loss function based on overlap measurement is less susceptible to fluctuations compared to weighted cross-entropy. Therefore, the Dice coefficient was utilized to focus on different tumor subregions. The formula for the Dice coefficient is given by:


(2) $$L_{\text{Dice}}=1-\frac{2\sum_{i=1}^{N}p_{i}g_{i}+\epsilon }{\sum_{i=1}^{N}p_{i}+\sum_{i=1}^{N}g+\epsilon }$$

In this formula, $g_{i}$ represents the ground truth label for pixel *i* in category *c*, and $p_{i}$ denotes the predicted probability of pixel *i* belonging to category *l*. The term *N* represents the total number of pixels in the image, and *ϵ* is a small constant added to avoid division by zero, ensuring the stability of the loss function.

For multi-class segmentation tasks, a weight $w_{l}$ is typically introduced based on the frequency of each category *l*. According to the statistical analysis of the proportion of each category, the weights were set to 0.1, 1, 2, and 2 for the background, WT, TC, and ET, respectively. The Multi-class Generalized Dice Loss (Multi-GDL) was then used as the model’s loss function, which can be written as:


(3) $$L_{\text{GDL}}=1-\frac{2\sum_{l=1}^{L}w_{l}\sum_{i=1}^{N}p_{i}^{\left(l\right)}g_{i}^{\left(l\right)}+\epsilon }{\sum_{l=1}^{L}w_{l}\left(\sum_{i=1}^{N}p_{i}^{\left(l\right)}+\sum_{i=1}^{N}g_{i}^{\left(l\right)}\right)+\epsilon }$$

In the above formula, *L* represents the total number of classes, and $p_{i}^{\left(l\right)}$ and $g_{i}^{\left(l\right)}$ denote the predicted probability and ground truth label for pixel *i* in class *l*, respectively. The weight $w_{l}$ ensures that the contribution of each class is appropriately adjusted based on its frequency, addressing the issue of data imbalance.

### 3.5 Postprocessing

**Figure 5:**
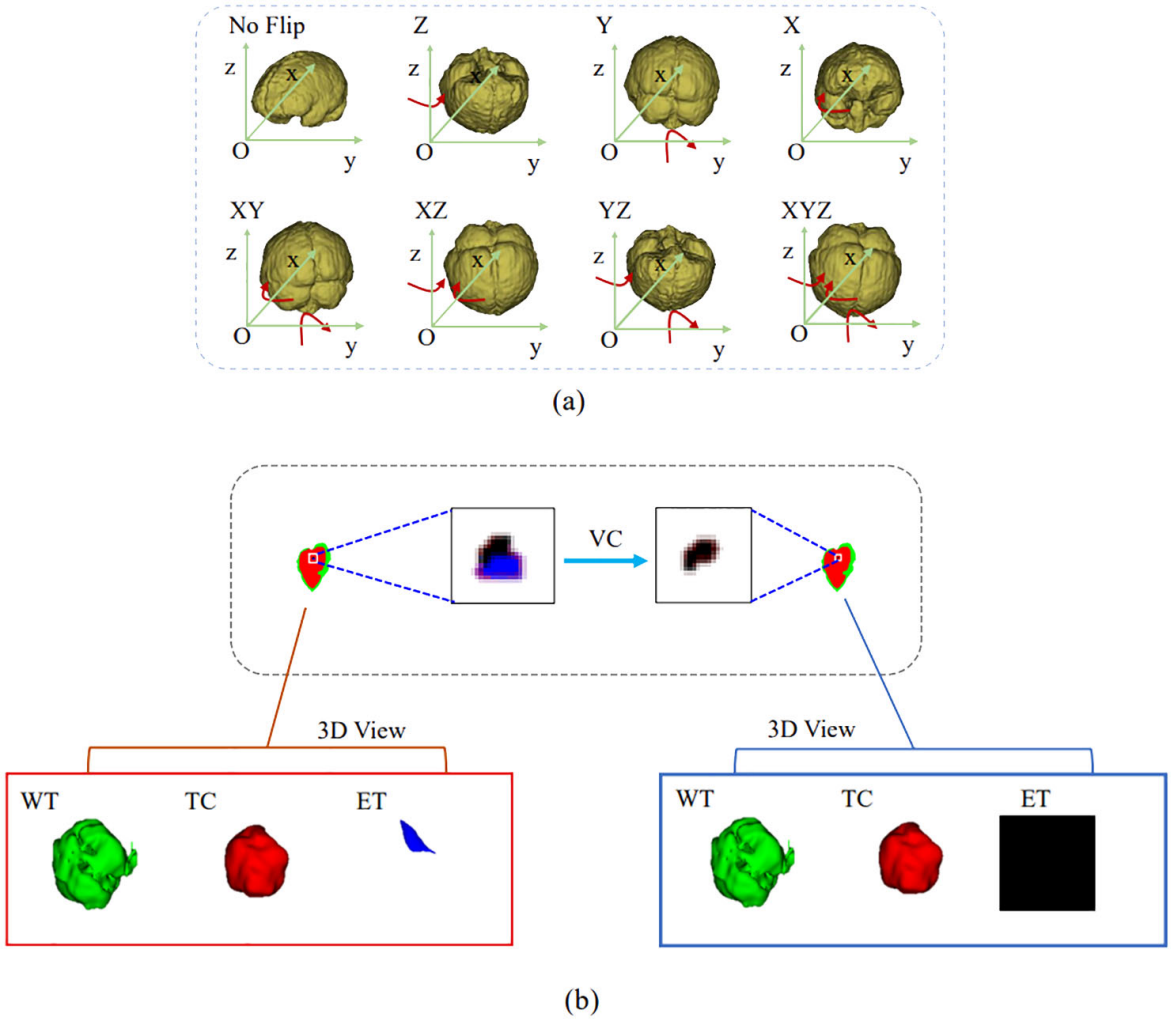
Results obtained by the postprocessing: **(A)** TTA; **(B)** VC. TTA was performed using 7 different flips: (x), (y), (z), (x, y), (x, z), (y, z), (x, y, z). VC replaces the ET predicted by the model if its volume is below the 500-voxel threshold.

In the inference phase, the original image was sliced from left to right and from top to bottom into eight inference blocks of size 128×128×128 and post-processed with test-time augmentation (TTA) and volume-constraint (VC). For each inference block, seven different flips ((*x*), (*y*), (*z*), (*x*, *y*), (*x*, *z*), (*y*, *z*), (*x*, *y*, *z*)) were performed, as shown in **Figure 5A**. The flipped data were then fed into the model, and the corresponding inference results were obtained. The rotation angles of the different inference results were restored, and the average value was taken as the final output.

For VC, if the reference segmentation for the ET is missing, the BraTS evaluation assigns a reward of 0 for false positive predictions, and the Dice score is 1. Therefore, in this study, if the ET volume predicted by the model was less than the threshold of 500, the ET region was reclassified as necrotic and non-enhanced tumor tissue. As shown in **Figure 5B**, the ET region is replaced with necrotic and non-enhanced tumors after volume restriction.

## 4 Experimental setting

### 4.1 Dataset

Three public benchmark datasets and one private dataset were used to evaluate the effectiveness of the proposed DeepGlioSeg. The Brain Tumor Segmentation Challenge provided the BraTS2019, BraTS2020, and BraTS2021 datasets used in this study (6, 34, 35). The BraTS2019 dataset consists of 335 training cases and 125 validation cases. The BraTS2020 dataset contains 369 training examples and 125 validation examples. The BraTS2021 dataset includes 1251 training samples and 219 validation samples.

The ZZH dataset was collected by the first affiliated hospital of Zhengzhou University with institutional review board approval (reference number: 2019-KY-231). It consists of 232 patient records in the same format as the BraTS datasets. Each sample was manually labeled by two radiologists at the first affiliated hospital of Zhengzhou University. The dataset was split into training, validation, and test sets in a 7:1:2 ratio. The training set was used to train the model, the validation set was used to guide hyperparameter tuning and early stopping, and the test set was used to evaluate generalization. This approach prevents data leakage and ensures an unbiased performance evaluation.

### 4.2 Evaluation metrics

The Dice similarity coefficient (Dice), Sensitivity (Sen), and Hausdorff distance (Haus95) are used to assess the segmentation performance of the model. Considering the glioma’s anatomical features and structure, the model’s performance in segmenting the following three tumor sub-regions is evaluated: WT (necrotic and non-enhanced tumor, peritumoral edema, and enhanced tumor), TC (necrotic and non-enhanced tumor, enhanced tumor), and ET (enhanced tumor).

### 4.3 Experimental details

The optimization method used is Adam with a learning rate of 0.0002, and training is performed with a batch size of 4. DeepGlioSeg is trained for approximately 1000 iterations. A minimum loss value threshold is set, and the average loss value of each epoch is calculated during training. Training is stopped when the loss value drops below the set threshold. The size of the image input to the model is 128×128×128×4.

After each downsampling layer, the size of the feature map is halved, and the number of channels is doubled. The number of initial convolution kernels is 16. The loss weights for the four regions—ET, WT, TC, and background—are set to [2, 1, 2, 0.1]. The following data augmentation techniques are applied: (1) random cropping of the data from 240×240×155 to 128×128×128; (2) random mirroring and rotation in the axial, coronal, and sagittal planes with a probability of 0.5; and (3) random intensity shifts in the range [-0.1, 0.1] and scaling factors in the range [0.8, 1.2]. The network is trained using Multi-GDL, and L2 normalization is applied to regularize the model, with the weight decay rate set to 1e-5.

## 5 Results

### 5.1 Ablation study

#### 5.1.1 CTPC module configuration

We conducted experiments to identify the optimal configuration of the CTPC module for better segmentation performance. **Table 1** summarizes the impact of different CTPC configurations on model performance. The baseline model employs a standard encoder-decoder architecture with separate CNN and Transformer branches, which independently extract local and global features. Although effective individually, the lack of integration between these branches limits the model’s ability to combine local and global information, reducing segmentation accuracy.

**Table 1:** Qualitative comparison of results on the BraTS2020 dataset, including the model architecture without the CTPC module (Baseline), encoding path configuration (EPC), decoding path configuration (DPC), and encoding-decoding path configuration (EDPC).

	ET	Dice		Mean	ET	Sen		Mean	ET	Haus95		
		WT	TC			WT	TC			WT	TC	Mean
Baseline	0.770	0.895	0.728	0.798	0.783	0.899	0.695	0.792	41.7	6.25	26.1	24.7
EPC	0.753	0.892	0.854	0.835	0.775	0.906	0.770	0.817	32.8	6.80	12.9	17.5
DPC	0.753	0.895	0.858	0.835	0.781	0.914	0.799	0.831	36.5	6.88	10.1	17.8
EDPC	0.768	0.897	0.865	0.843	0.785	0.911	0.812	0.836	27.1	5.92	9.94	14.3

Red denotes the best results, and blue means the second best.

DeepGlioSeg addresses this by incorporating the CTPC module, which enables the simultaneous fusion of local and global features. Unlike the baseline, where features are processed separately, the CTPC module integrates the outputs from both branches, fusing local and global features into a unified representation. This enhanced feature fusion improves segmentation accuracy, particularly for complex and heterogeneous tumor regions. The fully embedded CTPC model achieved a 4.5% improvement in Dice score (84.3% vs. 79.8%) over the baseline, demonstrating the effectiveness of this integration.

The CTPC module addresses challenges in feature alignment and compatibility between CNN and Transformer outputs. By using 1×1×1 convolutions for channel alignment, it ensures CNN features match the Transformer input dimensions, preserving local detail while facilitating global feature integration. Downsampling reduces the volume dimensions to 16×16×16, balancing computational efficiency with feature richness for global context. The final reshaping generates patch embeddings that facilitate effective local-global interaction, making the CTPC module highly effective for capturing complex patterns, crucial for tumor segmentation tasks.

#### 5.1.2 Learnable position embedding

In the work of Dosovitskiy (36), a learnable embedding was incorporated into the embedded patch sequence and complemented with position embeddings to preserve critical positional information. Similarly, for glioma segmentation, we introduced a learnable position embedding to encode crucial positional information for the task. Within the CTPC module, the CNN and Transformer branches enable the fusion of feature streams through a shared pathway. Before passing the CNN feature stream into the Transformer branch, we used standard one-dimensional learnable position embeddings to encode position information. The embeddings were then added to the feature map via summation. As shown in **Table 2**, the introduction of learnable position embeddings improved the average Dice score by 1% (84.3% vs. 83.3%).

**Table 2:** Ablation study of the CTPC architecture on the BraTS2020 dataset, testing the impact of different components. .

	ET	Dice		Mean	ET	Sen		Mean	ET	Haus95		
		WT	TC			WT	TC			Mean	TC	Mean
Pool	0.760	0.889	0.850	0.833	0.782	0.912	0.804	0.833	28.8	8.89	9.13	15.6
Scov	0.761	0.892	0.856	0.836	0.781	0.907	0.808	0.832	27.3	6.56	9.21	14.4
Sconv+PE	0.768	0.897	0.865	0.843	0.785	0.911	0.812	0.836	27.1	5.92	9.94	14.3

Red denotes the best results, and blue means the second best.

#### 5.1.3 Strided convolution

As shown in **Figure 3**, downsampling the feature map from the CNN branch is necessary to achieve spatial dimension alignment. Peng et al. (37) used average pooling in the feature coupling unit for this purpose. However, pooling can filter out valuable information during downsampling. To mitigate this, we chose strided convolution as the downsampling module. Strided convolution enables multiple downsampling steps while facilitating further feature extraction by adjusting the step size. The network uses four resolution levels (128, 64, 32, 16) from top to bottom, with downsampling modules having step sizes of 8, 4, 2, and 1, respectively. To ensure computational consistency, we maintained the patch embedding size in the Transformer branch at 4096. As shown in **Table 2**, using strided convolution as the downsampling module within the FFP improved the average Dice score by 0.7% (84.3% vs. 83.6%).

#### 5.1.4 Postprocessing

During inference, we used a dual post-processing approach involving TTA and VC. We evaluated the impact of these strategies on segmentation performance through comparative experiments, summarized in **Table 3**. The combined use of both strategies led to a 3.5% improvement in the average Dice score (84.3% vs. 80.8%). Importantly, these strategies improved performance without introducing additional computational complexity. We calculated a p-value for this metric, which was less than 0.05, supporting this improvement.

**Table 3:** Effect of post-processing on segmentation performance on the BraTS2020 dataset, evaluating strategies such as no post-processing (None), only TTA, and a combination of TTA and VC.

	ET	Dice		Mean	ET	Sen		Mean	ET	Haus95		
		WT	TC			WT	TC			Mean	TC	Mean
None	0.694	0.880	0.850	0.808	0.695	0.905	0.806	0.802	45.8	9.22	11.9	22.3
TTA	0.728	0.897	0.865	0.830	0.726	0.911	0.812	0.816	39.3	5.92	9.94	18.4
TTA+VC	0.768	0.897	0.865	0.843	0.785	0.911	0.812	0.836	27.1	5.92	9.94	14.3

Red denotes the best results, and blue means the second best.

**Figure 6:**
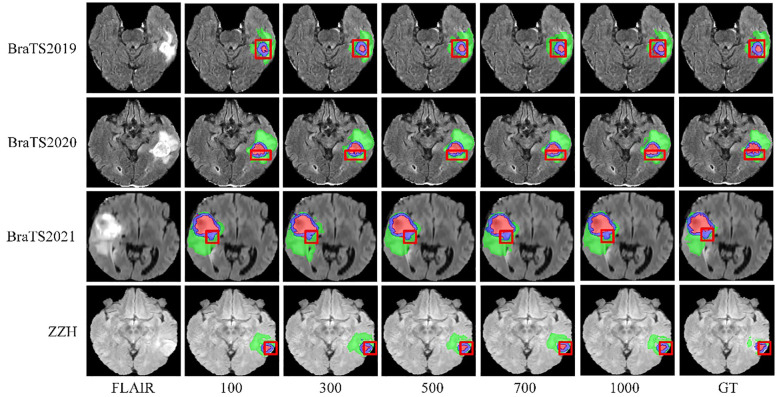
Visualization of results from different threshold selections in the VC post-processing technique on both the BraTS and ZZH datasets.

We systematically tested voxel count (VC) thresholds ranging from 100 to 1000 voxels to optimize the Dice score across tumor subregions, focusing on improving segmentation quality. As shown in **Figure 6**, a 500-voxel threshold achieved the best balance between false positives and true positives. At lower thresholds (e.g.,<500 voxels), over-segmentation occurred, leading to excessive false positives, particularly in the ET region, where small noise regions were incorrectly classified as tumor. Conversely, higher thresholds (>500 voxels) risked under-segmentation, excluding small but clinically significant tumor regions, reducing sensitivity and potentially missing subtle pathological features. The 500-voxel threshold effectively mitigated these issues, ensuring more robust and accurate segmentation across all tumor subregions.

To further justify this choice, we conducted a sensitivity analysis to evaluate the impact of different VC thresholds on segmentation performance. The results, summarized in **Table 4**, indicate that a threshold of 500 voxels consistently yielded the highest average Dice score while maintaining a favorable balance between precision and sensitivity across the four datasets. For the BraTS2019 dataset, the average Dice score reaches a maximum of 0.834 at a 500-voxel threshold, compared to 0.816 and 0.811 at thresholds of 100 and 1000, respectively. Sensitivity and precision also achieve their highest values of 0.838 and 0.842 at the 500-voxel threshold. In the BraTS2020 dataset, the average Dice score reaches a maximum of 0.843 when the threshold is 500, with sensitivity and precision also reaching their maximum values of 0.836 and 0.855, respectively. For the BraTS2021 dataset, when the threshold is 500, all evaluation metrics show excellent performance, with the average Dice score at 0.865, sensitivity at 0.855, and precision at 0.846. For the ZZH dataset, although all metrics are relatively low across all thresholds, at the 500-voxel threshold, the Dice coefficient, sensitivity, and precision are 0.616, 0.639, and 0.653, respectively, showing a relative advantage compared to the performance under other thresholds. Overall, setting the threshold at 500 for volume constraints generally yields better segmentation results.

**Table 4:** Comparison of segmentation performance with different thresholds across all the datasets.

Datasets	Threshold	Dice	Sensitivity	Precision
BraTS2019	100	0.816	0.833	0.835
	300	0.831	0.837	0.838
	500	0.834	0.838	0.842
	700	0.827	0.821	0.841
	1000	0.811	0.829	0.828
BraTS2020	100	0.821	0.825	0.828
	300	0.833	0.842	0.834
	500	0.843	0.836	0.855
	700	0.834	0.811	0.863
	1000	0.815	0.823	0.831
BraTS2021	100	0.827	0.824	0.822
	300	0.83	0.827	0.824
	500	0.865	0.855	0.846
	700	0.835	0.833	0.831
	1000	0.833	0.83	0.828
ZZH	100	0.574	0.603	0.621
	300	0.581	0.605	0.623
	500	0.616	0.639	0.653
	700	0.592	0.614	0.628
	1000	0.584	0.611	0.637

#### 5.1.5 Loss function

Brain tumor segmentation faces significant category imbalances, both between tumor and non-tumor tissue and among different tumor subregions. To address this, we assigned class weights based on category frequencies. Incorporating class weights into the GDL function increased the average Dice score by 5.2% (84.3% vs. 79.1%), as shown in **Table 5**, demonstrating its effectiveness in handling class imbalance.

**Table 5:** Comparison of segmentation performance with different loss functions on the BraTS2020 dataset.

	ET	Dice		Mean	ET	Sen		Mean	ET	Haus95		
		WT	TC			WT	TC			Mean	TC	Mean
DL	0.753	0.877	0.745	0.791	0.753	0.891	0.722	0.789	35.9	14.7	16.5	22.4
GDL	0.760	0.886	0.852	0.832	0.775	0.905	0.788	0.823	30.1	7.92	15.4	17.8
Multi-GDL	0.768	0.897	0.865	0.843	0.785	0.911	0.812	0.836	27.1	5.92	9.94	14.3

Red denotes the best results, and blue means the second best.

Glioma MRI datasets inherently exhibit imbalances among tumor regions, with the TC and ET being significantly smaller compared to the WT. To address this imbalance and emphasize clinically critical regions, we assigned higher weights to the ET and TC during training. Specifically, the model was configured with weights of 0.1 for the background, 1 for the WT, and 2 for the ET and TC, as shown in **Table 6**. This weighting strategy improved the Dice scores for the smaller regions by encouraging the model to prioritize them over the disproportionately large background and WT regions. We observed that increasing the weights for the ET and TC significantly enhanced their segmentation accuracy, ensuring better representation of these clinically significant areas. Simultaneously, reducing the background weight to 0.1 prevented the model from overfitting to irrelevant regions, which often dominate the data due to their larger size. Conversely, assigning higher weights to the background degraded the segmentation performance on the smaller regions, as the model became biased toward identifying the dominant background area. The selected weight configuration effectively strikes a balance by focusing on critical tumor subregions while minimizing distractions from the background, resulting in segmentation that is both accurate and clinically relevant.

**Table 6:** Comparison of segmentation performance with different weights using the GDL on all the datasets used.

Datasets	Weights	Dice	Sensitivity	Precision
BraTS2019	0.1, 1, 1, 1	0.802	0.812	0.817
	0.1, 1, 2, 2	0.834	0.838	0.842
	1, 1, 1, 1	0.795	0.801	0.812
	1, 1, 2, 2	0.799	0.806	0.814
BraTS2020	0.1, 1, 1, 1	0.831	0.822	0.838
	0.1, 1, 2, 2	0.843	0.836	0.855
	1, 1, 1, 1	0.805	0.793	0.812
	1, 1, 2, 2	0.812	0.806	0.825
BraTS2021	0.1, 1, 1, 1	0.844	0.835	0.833
	0.1, 1, 2, 2	0.865	0.855	0.846
	1, 1, 1, 1	0.833	0.827	0.822
	1, 1, 2, 2	0.841	0.835	0.828
ZZH	0.1, 1, 1, 1	0.596	0.616	0.635
	0.1, 1, 2, 2	0.616	0.639	0.653
	1, 1, 1, 1	0.574	0.583	0.591
	1, 1, 2, 2	0.586	0.615	0.633

#### 5.1.6 CNN branch and transformer branch

The CTPC module consists of two primary components: the CNN and Transformer branches. To better understand their contributions, we conducted ablation studies, with results summarized in **Table 7**. Removing the CNN branches caused a significant drop in segmentation performance, highlighting their critical role in the CTPC framework.

**Table 7:** Comparison of segmentation performance of the CNN branch and Transformer branch on the BraTS2020 dataset.

CNN branch	Trans branch	Mean Dice	Params
✗	✓	0.654	5.09M
✓	✗	0.798	5.47M
✓	✓	0.843	6.62M

Red denotes the best results, and blue means the second best.

In contrast, introducing the Transformer branch significantly improved performance at a relatively low parameter cost. This demonstrates the Transformer’s high efficiency and underscores its strength within the model.

#### 5.1.7 Different optimization strategy

We conducted experiments to evaluate the impact of different optimization strategies on model performance. Specifically, we tested SGD, Adam, and Adagrad on the BraTS2020 dataset to assess their influence on segmentation metrics. As summarized in **Table 8**, the SGD optimizer struggled with the ET metric (0.751), indicating difficulty in segmenting complex structures, though it performed slightly better on WT and TC metrics, averaging 0.811. In contrast, the Adam optimizer delivered the best overall performance, excelling in WT (0.897) and TC (0.865), demonstrating its ability to handle intricate segmentation tasks. Adagrad’s results were intermediate, performing well in WT (0.875) and TC (0.838) but falling short of Adam. These results highlight the need for effective optimizers like Adam to complement robust model architectures.

**Table 8:** Quantitative results comparing performance across different optimization strategy.

Strategy	Dice			
	ET	WT	TC	Mean
SGD	0.751	0.859	0.821	0.811
Adam	0.768	0.897	0.865	0.843
Adagrad	0.758	0.875	0.838	0.823

#### 5.1.8 Cross-dataset model testing

**Table 9** summarizes our evaluation of the model’s generalization, trained on BraTS2021 and tested on BraTS2019, BraTS2020, and ZZH datasets. On BraTS2019, the model achieved Dice scores of 0.645 (ET), 0.775 (WT), and 0.735 (TC), averaging 0.718. Performance improved slightly on BraTS2020, with scores of 0.651 (ET), 0.782 (WT), and 0.732 (TC), averaging 0.721, indicating good adaptation to consistent imaging protocols.

**Table 9:** Performance of cross-dataset model testing.

Cross Dataset	Dice			
	ET	WT	TC	Mean
BraTS2021->BraTS2019	0.645	0.775	0.735	0.718
BraTS2021->BraTS2020	0.651	0.782	0.732	0.721
BraTS2021->ZZH	0.411	0.705	0.431	0.515

In contrast, testing on the more heterogeneous ZZH clinical dataset resulted in lower Dice scores: 0.411 (ET), 0.705 (WT), and 0.431 (TC), averaging 0.515. This performance drop highlights the challenges of domain shifts and non-standardized imaging. These findings show the model’s robustness on standardized datasets but underline the need for domain adaptation to handle clinical variability.

### 5.2 Results

The comparison of the qualitative results for the BraTS2019, BraTS2020, BraTS2021, and ZZH datasets is displayed in **Table 10**. The segmentation outcomes for the three subregions on the BraTS datasets are similar, with WT achieving the highest accuracy and exhibiting fewer outliers. However, the annotation quality of the ZZH dataset for two subregions, ET and TC, could be improved.

**Table 10:** Qualitative comparison of results on the BraTS2019, BraTS2020, BraTS2021, and ZZH datasets.

Dataset	ET	Dice		Mean	ET	Sen		Mean	ET	Haus95		Mean
		WT	TC			WT	TC			WT	TC	
BraTS2019	0.761	0.887	0.854	0.834	0.785	0.905	0.809	0.836	33.2	7.03	7.09	15.8
BraTS2020	0.768	0.897	0.865	0.843	0.758	0.911	0.812	0.836	27.1	5.92	9.94	14.3
BraTS2021	0.808	0.91	0.878	0.865	0.836	0.925	0.843	0.868	22.4	5.06	10.7	12.7
ZZH	0.491	0.811	0.546	0.616	0.621	0.819	0.65	0.697	41.3	7.74	7.63	18.9

To further demonstrate the efficacy of the proposed DeepGlioSeg, nine advanced image segmentation algorithms in the field of medical image segmentation were reproduced, including 3D U-Net (38), 3D V-Net (39), Attention U-Net (40), nnU-Net (41), nnFormer (42), Segtran (43), SwinUNETR (44), TransBTS (45), and UNETR (23).

For the BraTS2019 dataset, the mean Dice scores for each method across the ET, WT, and TC regions, as well as the overall mean Dice score, are presented in **Table 11**. Notably, our proposed method demonstrates superior performance, achieving the highest Dice scores across all regions and the overall mean Dice score. Specifically, it achieves Dice scores of 0.761 for ET, 0.887 for WT, 0.854 for TC, and an impressive overall mean Dice score of 0.834. Conversely, other contemporary approaches exhibit varying levels of segmentation accuracy, with Dice scores ranging from 0.656 to 0.750 for ET, 0.831 to 0.879 for WT, 0.781 to 0.835 for TC, and 0.756 to 0.821 for the overall mean Dice score. These results underscore the significant improvement achieved by our proposed method over existing approaches, emphasizing its potential to advance the field of brain tumor segmentation.

**Table 11:** Quantitative results comparing the performance of our method with other state-of-the-art segmentation methods on the BraTS2019 dataset.

Method	Dice			
	ET	WT	TC	Mean
3DUnet	0.721	0.864	0.832	0.805
3DVnet	0.712	0.861	0.824	0.799
Atten-Unet	0.738	0.848	0.800	0.795
nnU-Net	0.741	0.868	0.834	0.814
nnformer	0.656	0.831	0.781	0.756
Segtran	0.725	0.858	0.831	0.804
SwinUNETR	0.750	0.879	0.835	0.821
TransBTS	0.741	0.862	0.834	0.812
UNETR	0.736	0.859	0.812	0.802
Proposed	0.761	0.887	0.854	0.834

Red denotes the best results, and blue means the second best.

For the BraTS2020 dataset, the results are summarized in **Table 12**. Our proposed method stands out as the best-performing approach, achieving the highest mean Dice scores: 0.897 for WT, 0.865 for TC, 0.768 for ET and 0.843 for the overall mean Dice score. It is important to highlight that SwinUNETR is the closest competitor to our proposed method, achieving remarkable mean Dice scores of 0.754 for ET, 0.883 for WT, 0.837 for TC, and 0.823 for the mean Dice score. Comparatively, while several other methods also show competitive performance, our proposed method consistently outperforms them across all regions, demonstrating its effectiveness in accurately segmenting brain tumors on the BraTS2020 dataset.

**Table 12:** Quantitative results comparing the performance of our method with other state-of-the-art segmentation methods on the BraTS2020 dataset.

Method	Dice			
	ET	WT	TC	Mean
3DUnet	0.746	0.870	0.848	0.821
3DVnet	0.734	0.865	0.844	0.814
Atten-Unet	0.742	0.852	0.802	0.818
nnU-Net	0.742	0.871	0.842	0.818
nnformer	0.659	0.833	0.785	0.759
Segtran	0.728	0.875	0.849	0.817
SwinUNETR	0.754	0.883	0.837	0.823
TransBTS	0.744	0.867	0.839	0.816
UNETR	0.734	0.864	0.817	0.813
Proposed	0.768	0.897	0.865	0.843

Red denotes the best results, and blue means the second best.

For the BraTS2021 dataset, **Table 13** provides a comprehensive overview of the segmentation performance of several methods. Our proposed method demonstrates exceptional segmentation accuracy, achieving the highest Dice scores across all regions. It stands out with Dice scores of 0.808 for ET, 0.910 for WT, 0.878 for TC, and an impressive mean Dice score of 0.865. This performance underscores the ability of our proposed method to accurately delineate brain tumor regions, indicating potential clinical relevance. SwinUNETR and Segtran also exhibit strong segmentation performance, achieving mean Dice scores of 0.854 and 0.845, respectively. Their robust performance reflects the effectiveness of their segmentation strategies and highlights their potential clinical utility. Our comprehensive evaluation on the BraTS2021 validation dataset confirms the exceptional performance of our proposed approach, outperforming all other methods across different segmentation regions.

**Table 13:** Quantitative results comparing the performance of our method with other state-of-the-art segmentation methods on the BraTS2021 dataset.

Method	Dice			
	ET	WT	TC	Mean
3DUnet	0.735	0.874	0.843	0.817
3DVnet	0.728	0.870	0.836	0.811
Atten-Unet	0.716	0.865	0.821	0.800
nnU-Net	0.764	0.887	0.852	0.834
nnformer	0.711	0.861	0.812	0.794
Segtran	0.772	0.896	0.869	0.845
SwinUNETR	0.782	0.905	0.875	0.854
TransBTS	0.761	0.885	0.850	0.832
UNETR	0.756	0.883	0.842	0.827
Proposed	0.808	0.910	0.878	0.865

Red denotes the best results, and blue means the second best.

To demonstrate the excellence of the proposed DeepGlioSeg in segmenting clinical datasets of suboptimal quality, we present the results of our experiments on the ZZH dataset, comparing the segmentation performance of several advanced technologies for brain tumor segmentation. **Table 5** provides a comprehensive evaluation of the segmentation effectiveness of nine advanced image segmentation technologies. From **Table 14**, we summarize the following key points: (1) The ZZH dataset presents unique challenges for brain tumor segmentation. The Dice scores of all methods are significantly lower compared to previous datasets, indicating the presence of complex tumor phenotypes and irregular shapes in this dataset. (2) Among the evaluated methods, our proposed method consistently achieves the highest Dice scores, demonstrating its effectiveness in addressing the challenges posed by the ZZH dataset. Specifically, it achieves an average Dice score of 0.616, indicating relatively strong segmentation performance even in this challenging context. (3) While our proposed method stands out, there is variability in the performance of other methods. SwinUNETR also shows competitive effectiveness, with an average Dice score of 0.613.

**Table 14:** Quantitative results comparing the performance of our method with other state-of-the-art segmentation methods on the ZZH dataset.

Method	Dice			
	ET	WT	TC	Mean
3DUnet	0.473	0.745	0.495	0.571
3DVnet	0.446	0.718	0.506	0.556
Atten-Unet	0.430	0.731	0.461	0.540
nnU-Net	0.486	0.808	0.531	0.608
nnformer	0.419	0.701	0.488	0.536
Segtran	0.452	0.804	0.573	0.609
SwinUNETR	0.506	0.803	0.531	0.613
TransBTS	0.434	0.768	0.491	0.564
UNETR	0.441	0.735	0.482	0.552
Proposed	0.491	0.811	0.546	0.616

Red denotes the best results, and blue means the second best.

The lower segmentation performance on the ZZH dataset compared to the BraTS datasets arises from differences in data quality, diversity, imaging protocols, and real-world complexities. First, BraTS benefits from high-quality, standardized annotations by multiple radiologists, ensuring consistent labels. In contrast, ZZH annotations reflect varying expertise and subjective judgments, introducing inconsistencies, particularly for smaller regions like ET. Second, the BraTS datasets are diverse, encompassing varied patient demographics, tumor grades, and imaging conditions, enabling better generalization. By comparison, ZZH’s limited and less diverse sample restricts feature learning and introduces potential demographic biases. Third, standardized imaging protocols in BraTS ensure consistent data characteristics, whereas ZZH exhibits variability in scanner models, field strengths, and acquisition parameters, affecting tumor visibility and segmentation accuracy. Lastly, ZZH reflects real clinical challenges, such as artifacts, motion blur, and non-standardized conditions, which are less prevalent in BraTS.

**Figure 7:**
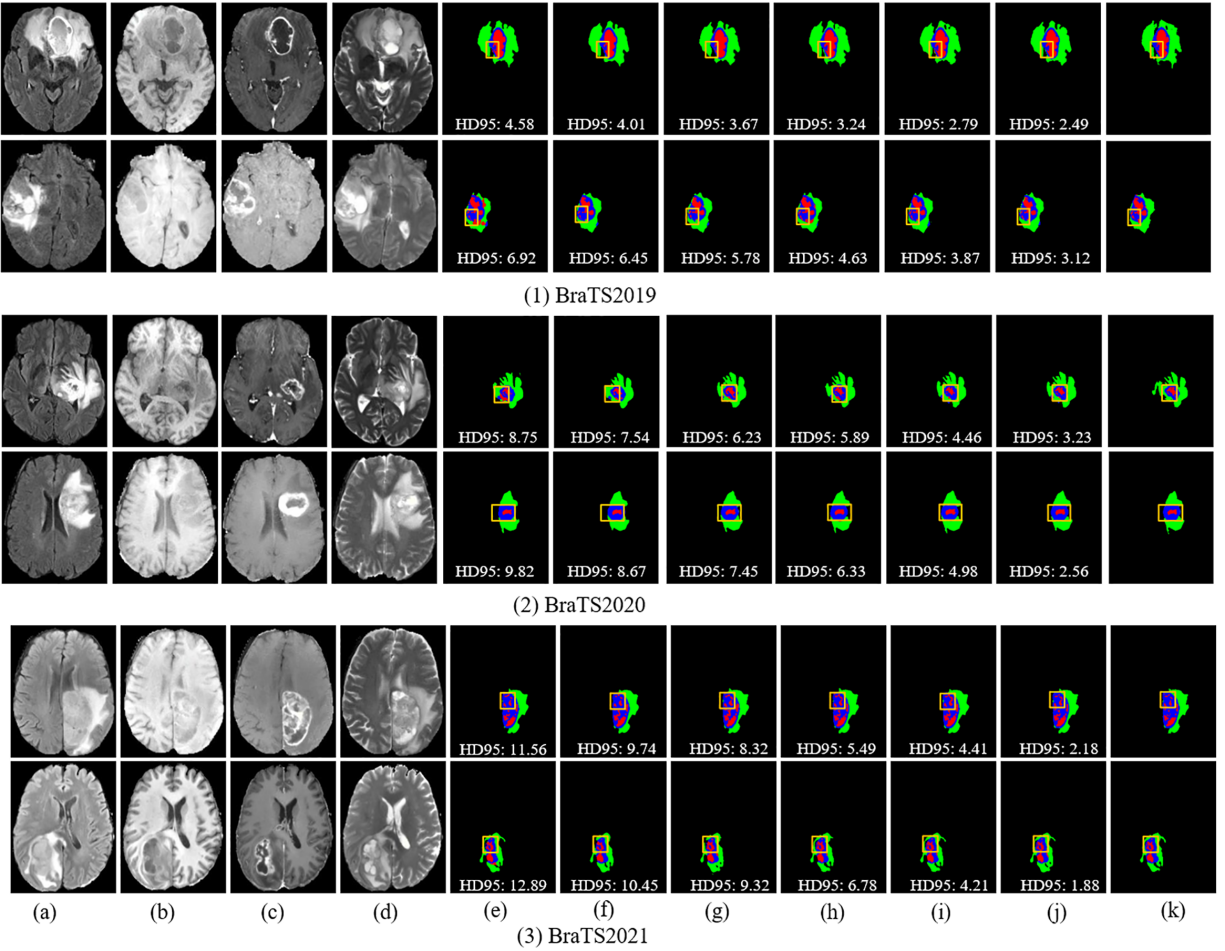
Visual comparison of segmentation results with different models on the BraTS2019, BraTS2020, and BraTS2021 datasets. **(A)** FLAIR. **(B)** T1. **(C)** T1-CE. **(D)** T2. **(E)** Attention-Unet. **(F)** nnU-Net. **(G)** Segtran. **(H)** SwinUNETR. **(I)** TransBTS. **(J)** Ours. **(K)** GT.

**Figure 8:**
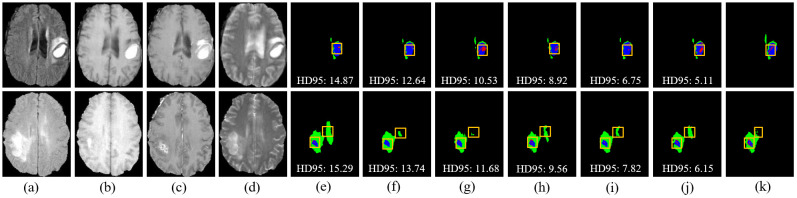
Visual comparison of segmentation results with different models on the ZZH dataset. **(A)** FLAIR. **(B)** T1. **(C)** T1-CE. **(D)** T2. **(E)** Attention-Unet. **(F)** nnU-Net. **(G)** Segtran. **(H)** SwinUNETR. **(I)** TransBTS. **(J)** Ours. **(K)** GT.

**Figures 7** and **8** provide visual comparisons of segmentation results from various methods on the BraTS2019, BraTS2020, and BraTS2021 datasets, supported by quantitative metrics such as HD95 and boundary overlap to objectively assess boundary quality. The HD95 metric, which evaluates worst-case boundary deviations, highlights the precision of DeepGlioSeg compared to state-of-the-art models such as SwinUNETR and Segtran. For instance, DeepGlioSeg achieves an HD95 of 5.09 for the ET on BraTS2021, outperforming SwinUNETR (6.45) and Segtran (6.78), indicating superior boundary alignment. Additionally, boundary overlap metrics such as Dice scores reinforce these findings. DeepGlioSeg achieves a Dice score of 0.92 for the WT, outperforming models that struggle with under-segmentation or over-segmentation in intricate regions. This performance is attributed to DeepGlioSeg’s hybrid CNN-Transformer architecture, which effectively integrates local detail extraction and global context modeling, enabling precise tumor boundary delineation even in challenging cases. These quantitative results align with the visual comparisons, demonstrating DeepGlioSeg’s capability to handle complex boundary variations.

**Figure 9:**
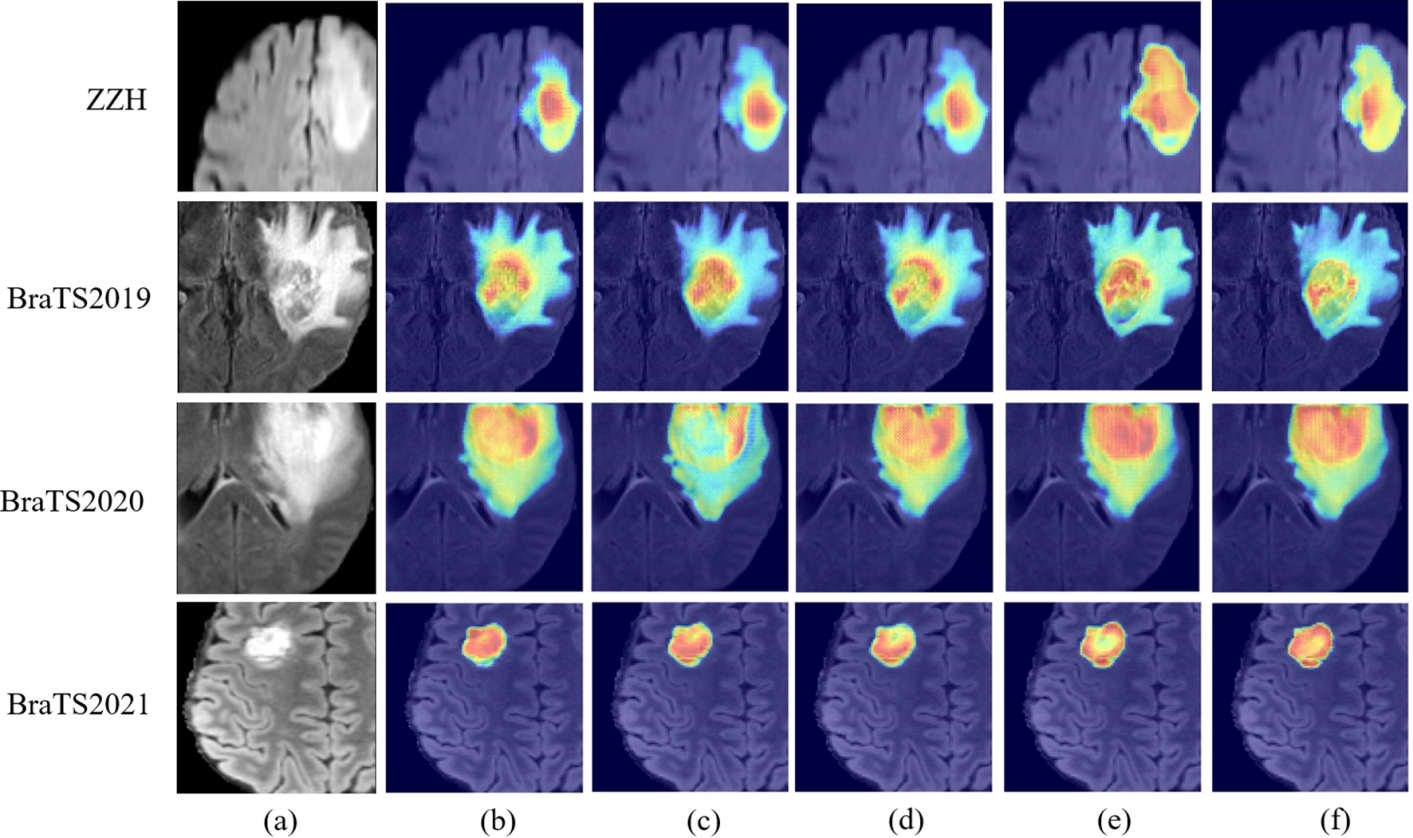
Visual comparison of feature maps with different models on the ZZH, BraTS2019, BraTS2020, and BraTS2021 datasets. **(A)** Raw image. **(B)** nnU-Net. **(C)** Segtran. **(D)** SwinUNETR. **(E)** Ours. **(F)** TransBTS.

However, challenges persist in tumors with high heterogeneity, where significant variability in appearance affects segmentation consistency. DeepGlioSeg demonstrates superior performance in handling such heterogeneity, achieving consistent results across various datasets, such as ZZH, BraTS2019, BraTS2020, and BraTS2021. Its success lies in the flexible CTPC architecture, which integrates CNNs for capturing localized details and Transformers for modeling global context. This dual-pathway approach enables the seamless fusion of local and global features, ensuring accurate delineation of complex tumor boundaries. As highlighted in **Figure 9**, DeepGlioSeg produces sharper and more precise tumor segmentations than nnU-Net and SwinUNETR, particularly in real-world datasets (e.g., ZZH), which exhibit greater variability than more standardized datasets such as BraTS.

DeepGlioSeg’s design directly overcomes the limitations of existing models. nnU-Net, while efficient in capturing local features, struggles to generalize across datasets due to its lack of global context modeling. SwinUNETR incorporates Transformers for global feature representation but lacks the balanced local-global integration of DeepGlioSeg, limiting its ability to segment tumors with complex boundaries in heterogeneous datasets. In contrast, the CTPC module’s efficient fusion of local and global features allows DeepGlioSeg to excel in identifying subtle tumor variations. This capability is crucial for accurate segmentation in real-world clinical settings.

## 6 Discussion

Gliomas present significant challenges for segmentation due to their complex heterogeneity, including variability in shape, structure, and location. Accurate tumor boundary delineation is essential, requiring models capable of capturing both local and global features. However, many existing methods struggle to achieve this integration effectively. To address this challenge, we propose the DeepGlioSeg framework, which integrates a CTPC module with parallel CNN and Transformer branches. The CNN branch captures fine-grained local details, while the Transformer branch models long-range dependencies. The combination of these two pathways ensures a robust fusion of local and global features, which enhances the model’s ability to represent complex tumor characteristics—such as varying shapes and structural heterogeneity—resulting in improved segmentation accuracy.

In addition to effective feature fusion, DeepGlioSeg tackles the issue of class imbalance—a common problem in glioma segmentation, where certain tumor regions, such as the ET, are underrepresented. To address this, we employ a weighted loss function that extends the generalized Dice loss. By assigning higher weights to clinically significant but underrepresented regions (like ET), the model can prioritize these areas during training, ensuring accurate segmentation of both larger regions (e.g., the WT) and smaller, more challenging regions crucial for treatment planning. This approach helps mitigate the bias toward larger regions, which is often seen in conventional models.

**Table 15** compares the computational complexity of the proposed method with state-of-the-art models across four datasets: BraTS2019, BraTS2020, BraTS2021, and ZZH. Key metrics include the number of parameters (Params in M), computational cost (FLOPs in G), and statistical significance (p-value). Transformer-based models, such as TransBTS and SwinUNETR, exhibit higher parameter counts (15.56M–30.62M) and computational costs (163.73G–254.45G), while lighter models like 3DUNet and Atten-Unet maintain smaller parameter sizes but incur higher FLOPs. The proposed method strikes an optimal balance with 6.92M parameters and 156.79G FLOPs, significantly reducing computational demands while maintaining competitive performance. Lower p-values across datasets confirm the statistical significance of the proposed method’s improvements, emphasizing its efficiency and robustness compared to other models.

**Table 15:** Comparison of computational complexity between our method and other state-of-the-art methods on all the datasets used.

Datasets	Method	Params (M)	FLOPS (G)	*p*-value
BraTS2019	3DUnet	5.42	275.53	2e-6
	3DVnet	4.76	157.81	4e-5
	Atten-Unet	2.47	164.06	2e-4
	nnU-Net	9.4	84.03	0.0012
	nnformer	14.91	172.04	0.0048
	Segtran	29.19	254.45	0.0035
	SwinUNETR	15.56	206.76	0.0072
	TransBTS	30.62	163.73	0.0223
	UNETR	15.56	206.76	0.0185
	Proposed	6.92	156.79	*
BraTS2020	3DUnet	5.42	275.53	1e-7
	3DVnet	4.76	157.81	3e-4
	Atten-Unet	2.47	164.06	1e-4
	nnU-Net	9.4	84.03	0.0015
	nnformer	14.91	172.04	0.0053
	Segtran	29.19	254.45	0.0041
	SwinUNETR	15.56	206.76	0.0073
	TransBTS	30.62	163.73	0.0244
	UNETR	15.56	206.76	0.0157
	Proposed	6.92	156.79	*
BraTS2021	3DUnet	5.42	275.53	1e-7
	3DVnet	4.76	157.81	3e-6
	Atten-Unet	2.47	164.06	1e-5
	nnU-Net	9.4	84.03	0.0009
	nnformer	14.91	172.04	0.0052
	Segtran	29.19	254.45	0.0032
	SwinUNETR	15.56	206.76	0.0068
	TransBTS	30.62	163.73	0.0277
	UNETR	15.56	206.76	0.0136
	Proposed	6.92	156.79	*
ZZH	3DUnet	5.42	275.53	1e-8
	3DVnet	4.76	157.81	5e-4
	Atten-Unet	2.47	164.06	6e-4
	nnU-Net	9.4	84.03	0.0018
	nnformer	14.91	172.04	0.0046
	Segtran	29.19	254.45	0.0037
	SwinUNETR	15.56	206.76	0.0076
	TransBTS	30.62	163.73	0.0218
	UNETR	15.56	206.76	0.0165
	Proposed	6.92	156.79	*

The p-value is computed for paired t-tests between our method and other methods. A p-value less than 0.05 indicates the statistical significance of the paired t-tests.

The symbol * indicates that the p-values for the other methods were calculated using a paired samples t-test, with our method serving as the benchmark reference.

Beyond glioma segmentation, the CTPC module is highly adaptable and could be applied to other medical imaging tasks. Its ability to integrate local and global features makes it well-suited for segmenting tumors with irregular boundaries, such as lung or liver tumors. Additionally, the framework supports multi-modal imaging (e.g., PET-CT, MRI-CT fusion), allowing the model to combine complementary information for more accurate segmentation. In the future, we plan to enhance the model further by incorporating dynamic attention-based feature selection and task-specific fusion strategies, broadening its clinical applicability.

Despite these strengths, the model is not without limitations. The complexity and large parameter count increase the risk of overfitting, especially when trained on smaller datasets. To mitigate this, regularization techniques such as dropout, data augmentation, and early stopping could improve the model’s robustness. Another challenge is the variability in MRI data from different scanners or imaging protocols, which can hinder generalization. Future work will focus on data harmonization methods, such as domain adaptation and intensity normalization, to reduce these challenges and improve generalization across diverse datasets.

Looking ahead, there are several opportunities for further refinement of DeepGlioSeg. Incorporating multi-scale feature extraction, attention mechanisms, and multi-task learning can enhance its ability to handle a broader range of clinical tasks. Additionally, transfer learning from pre-trained models and the inclusion of contextual priors could reduce dependency on large labeled datasets, improving the model’s adaptability to various imaging modalities and expanding its clinical utility.

## 7 Conclusion

In this study, we present DeepGlioSeg, a novel framework developed to address the challenging task of automating brain tumor segmentation. Our proposed method incorporates the CTPC module into an encoder-decoder network architecture, enabling it to capture critical local features of gliomas, such as texture and edges. To tackle the challenge of category imbalance, we introduce the Multi-GDL loss function, which adjusts category weights to rebalance loss contributions, resulting in more accurate identification of tumor structures. To further enhance glioma segmentation during inference, we employ a combination of TTA and VC as post-processing strategies. These improvements highlight the effectiveness of the CTPC module, Multi-GDL loss function, and post-processing strategies. Future enhancements for segmenting complex regions like ET and TC may involve refining the CTPC module with dynamic attention-based feature fusion to adaptively focus on intricate boundaries. Additionally, multi-scale learning and adaptive weighted loss functions could further improve segmentation by capturing multi-resolution features and prioritizing critical regions.

## Data Availability

Publicly available datasets were analyzed in this study. This data can be found here: The datasets utilized in this study, including BraTS2019, BraTS2020, and BraTS2021, are publicly accessible at the following URLs: BraTS2019: https://www.med.upenn.edu/cbica/brats-2019/ BraTS2020: https://www.med.upenn.edu/cbica/brats2020 BraTS2021: https://www.med.upenn.edu/cbica/brats2021 However, the private dataset referred to as ZZH, which is also discussed in this paper, is currently not publicly available. The raw and processed data required to replicate these study results are part of an ongoing research project and cannot be shared at this time. For inquiries regarding access to the ZZH dataset, please contact RL at drlrp2022@163.com.
